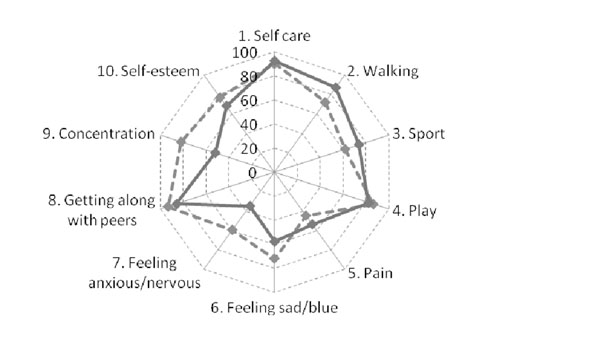# Children with juvenile idiopathic arthritis currently followed in a tertiary care setting have a better psychosocial well-being than healthy peers

**DOI:** 10.1186/1546-0096-9-S1-O22

**Published:** 2011-09-14

**Authors:** M Bertamino, A Consolaro, B Lattanzi, S Magni-Manzoni, S Lanni, C Suffia, S Dalprà, S Rosina, A Martini, A Ravelli

**Affiliations:** 1 Pediatria II, IRCCS G.Gaslini, Genova,Italy; 2 Dipartimento di Pediatria, Università degli Studi di Genova, Italy; 3 Clinica Pediatrica, Fondazione IRCCS Policlinico S. Matteo, Pavia, Italy

## Background

Assessment of health-related quality of life (HRQL) is a fundamental component of the clinical evaluation of children with pediatric rheumatic diseases. However, comparison with healthy children (HC) has seldom been attempted.

## Aim

To compare the HRQL of children with juvenile idiopathic arthritis (JIA) with that of HC.

## Methods

669 parents of children with JIA, 398 children with JIA, 801 parents of HC, and 796 HC completed independently the Pediatric Rheumatology Quality of Life scale (PRQL) (Filocamo et al. Rheumatology 2010). Children with JIA and HC who completed the questionnaire were aged > 7-8 years. The PRQL is a 10-item questionnaire that includes 2 subdimensions, physical health (PhH) and psychosocial health (PsH), each composed of 5 items. The total PRQL score ranges from 0 to 30, with higher scores indicating worse HRQOL. A separate score for the PhH and PsH subscales (range 0–15) can be calculated.

## Results

**Figure 1:**
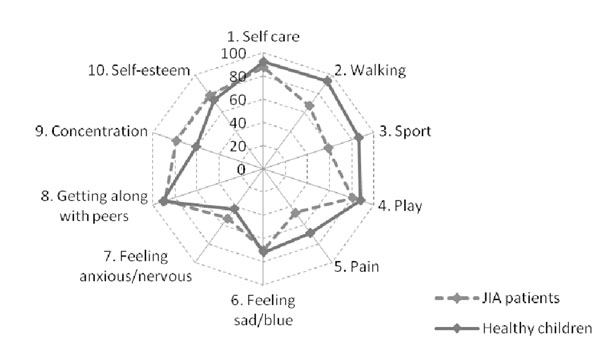


The median PRQL total score in children with JIA and HC was comparable for both parent proxy-reports (2 and 2, respectively) and child self-reports (2 and 3, respectively). The frequency of 0 scores (=normal HRQL) for the total PRQL score was higher in children with JIA than in HC for both parent proxy-reports (25.9% and 22.8%, respectively) and child self-reports (23.4% and 11.7%, respectively). However the frequency of 0 scores for the PhH subscale was greater in HC than in children with JIA for both parent proxy-reports (55.1% and 36.4%, respectively) and child self-reports (37.8% and 36.8%, respectively). Unexpectedly, the frequency of 0 scores for the PsH subscale was greater in children with JIA than in HC for both parent proxy-reports (43%% and 27.5%, respectively) and child self-reports (44.2% and 17.5%, respectively). Figures 1 and 2 show the frequency of 0 scores for the 10 items of the PRQL, with items 1-5 referring to PhH and items 6-10 referring to PsH. PhH items more frequently impaired in JIA patients walking, sports, and pain.

## Conclusion

To our knowledge, our study is the first to show that psychosocial well-being of children with JIA is better than that of HC. This phenomenon may depend, at least partially, on most of the JIA patients currently attending tertiary care hospitals for follow-up visits having well-controlled disease with little or no disease activity or disability. It is conceivable that relief of active disease symptoms leads to a marked enhancement in their mental and social health. This observation deserves further exploration in different populations.

**Figure 2:**